# Electrocardiographic Predictors of Major Adverse Cardiovascular Events in Women With Suspected Ischemia and no Obstructive Coronary Artery Disease: Results of the Women's Ischemia Syndrome Evaluation

**DOI:** 10.1111/anec.70168

**Published:** 2026-03-28

**Authors:** Jennifer R. Dungan, Yasmeen K. Taha, Qinglin Pei, Michael T. Weaver, Michael J. Tavormina, Steven E. Reis, Eileen M. Handberg, C. Noel Bairey Merz, Carl J. Pepine

**Affiliations:** ^1^ Division of Cardiovascular Medicine, College of Medicine University of Florida Gainesville Florida USA; ^2^ Biobehavioral Nursing Science Department, College of Nursing University of Florida Gainesville Florida USA; ^3^ UF Health Shands Hospital Thoracic Surgical & Lung Transplant Unit Gainesville Florida USA; ^4^ University of Pittsburgh Medical Center Pittsburgh Pennsylvania USA; ^5^ Barbra Streisand Women's Heart Center, Smidt Heart Institute Cedars‐Sinai Medical Center Los Angeles California USA

**Keywords:** electrocardiography, female, ischemia and no obstructive coronary arteries, major adverse cardiac events, mortality, open artery ischemia, women

## Abstract

**Introduction:**

An estimated four million individuals have suspected Ischemia and No Obstructive Coronary Arteries (INOCA), often due to coronary microvascular dysfunction, a subtype of ischemic heart disease that disproportionately affects women. Previously hypothesized as a benign condition, patients with INOCA demonstrate an elevated risk profile for major adverse cardiovascular events (MACE), yet biomarkers to inform this risk have not been identified.

**Methods:**

To examine ECG indices as predictors of MACE among women with INOCA, we conducted a secondary analysis of the Women's Ischemia Syndrome Evaluation (WISE) original cohort in 481 women with suspected INOCA and complete data. Cox multivariable regression analysis was conducted in baseline ECG indices (intervals, rate, rhythm, and abnormalities) and their association with time‐to‐incident MACE (angina hospitalization, myocardial infarction, stroke, heart failure, revascularization with stent or angioplasty, or all‐cause death). Cox proportional hazards modeling was performed as time from study enrollment to event censored on days to last contact, using stepwise backward selection and bootstrapping for model determination.

**Results:**

Overall, 165 (34.3%) experienced at least one MACE, including 26 (5.41%) all cause deaths, over an average follow‐up time of 5.25 years. Significant predictors in the final model were resting heart rate (RHR), race, CHF, depression, and use of nitrates within 24 h of ECG measurement. Higher RHR was associated with increased hazards of incident composite MACE (adjusted hazard ratio 1.018, *p* = 0.0348).

**Conclusion:**

Identifying unique biomarkers for MACE among women with INOCA is a clinical and research priority to inform screening and prevention strategies.

## Graphical abstract

A cohort of 481 female participants of the Women's Ischemia Syndrome Evaluation (WISE) Original study clinically phenotyped as having suspect ischemia with no obstructive coronary arteries (INOCA) were evaluated for baseline ECG and clinical predictors of time to incident major adverse cardiac events (MACE). Formerly considered a benign condition, an estimated 34% of females with INOCA experienced at least one MACE event across an average of just over 5 years, independently predicted by resting heart rate (RHR), race, congestive heart failure (CHF), depression, and use of nitrates within 24 h of ECG measurement.

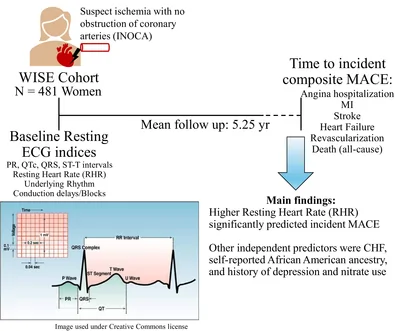

## 1 Introduction

Coronary open artery ischemia syndromes contribute to major adverse cardiac events (MACE), similar to obstructive disease (Lindahl et al. 2017). Ischemia with no obstructive coronary arteries (INOCA) is a type of open artery myocardial ischemia defined as the presence of less than 50% stenosis of coronary blood vessels (Hansen et al. 2023). INOCA is more common in women with an estimated 60% prevalence among those who are symptomatic (Herscovici et al. 2018; Pepine 2023). Because of the lack of majorly stenosed coronary arteries, INOCA was generally considered a benign risk for serious cardiovascular complications (Herscovici et al. 2018). Original WISE study data indicate that INOCA patients are at an intermediate risk for MACE at a rate of 2.5% each year by 5 years after their initial diagnosis (Bairey Merz et al. 2017). After 10 years, 20% of WISE cohort INOCA patients had died, with 62% of the deaths attributable to a cardiac cause (Pepine 2023), despite having an intermediate Atherosclerotic Cardiovascular Disease (ASCVD) risk profile (Bairey Merz et al. 2017), estimates that are consistent with that of other reports (Herscovici et al. 2018). Most stable patients with INOCA are classified as having an intermediate Framingham Risk Score for MACE occurrence (10%–20% risk of 10‐year events), even when micro and macrovascular dysfunction are included in the risk score determination (Herscovici et al. 2018; Reriani et al. 2016). This suggests that MACE disparities among females with INOCA are underestimated by current risk scores. Moreover, misconceptions increase the risk of improper clinical management for those with INOCA (Mehta et al. 2022). Given the prevalence and elevated risk of INOCA in women for MACE, clinical predictors, biomarker identification and MACE prevention strategies are clinical priorities.

A major clinical source of core indicators for myocardial ischemia in the presence of symptoms is electrocardiography (ECG). In a systematic review, resting ECG was the most useful bedside test for myocardial infarction (MI) (Chun and McGee 2004), and ECG measures predicting MACE and mortality are well established in studies of various non‐INOCA contexts (Brenyo and Zareba 2011; Cheng et al. 2009; Kashani and Barold 2005; Kwok et al. 2016; O'Neal et al. 2017; Welten, Elders, et al. 2023; Welten, van der Heijden, et al. 2023).

The Women's Ischemia Syndrome Evaluation (WISE) cohort study (Merz et al. 1999) has laid substantial groundwork in the study of sex‐associated INOCA and outcomes (Barsky et al. 2020). An early evaluation of data from 143 WISE participants who had deep phenotyping of sophisticated ECG parameters demonstrated that, along with CAD severity, QRS‐T angle, wider QRS complex, QTrr, and a more depressed ST‐segment in precordial lead V′_5_ were independent predictors of mortality, CHF, and non‐fatal myocardial infarction (MI) (Triola et al. 2005). At WISE study completion, an evaluation of 904 participants identified that a longer corrected QT (QTc) interval was an independent predictor of sudden cardiac death among the full sample, including when subset for INOCA (*n* = 551) (Mehta et al. 2017). However, these studies specifically focused on women with preserved ejection fraction and a limited set of MACE outcomes. A more recent analysis of WISE participants with INOCA evaluated MACE outcomes between women with negative vs. positive dobutamine stress ECG (> 1 mm ST change) in those without wall motion abnormalities (*N* = 82) (Ranasinghe et al. 2023). The CIAO‐ISCHEMIA Study reported ECG predictors of MACE among men and women with INOCA but failed to report sex differences (Reynolds et al. 2021). Given these former findings, the purpose of this study was to evaluate whether readily available resting ECG indices could predict composite, incident MACE outcomes among the entire sample of symptomatic women with suspect INOCA in the WISE cohort.

## 2 Materials and Methods

The present project was approved by the University of Florida Institutional Review Board (IRB; Protocol #IRB202102834). We conducted a secondary analysis of the original WISE study biobank completed in 1999 (Protocol #IRB201501099). WISE screened 7603 women and enrolled 936, ranging from ages 18–84, at the University of Florida, University of Pittsburgh, Allegheny General Hospital in Pittsburgh, Cedars‐Sinai Medical Center, and at the University of Alabama at Birmingham (Merz et al. 1999). Participants over 18 with ischemic symptoms underwent coronary angiography to assess their coronary arteries and determine if they had an MI (Merz et al. 1999). In a stepwise manner, the recruitment process included checking inclusion and exclusion criteria, completing screening questions, collecting blood samples, acquiring resting/ambulatory ECGs, and then completing coronary angiography (Merz et al. 1999). Suspected INOCA was defined as < 50% stenosed coronary arteries in the presence of ischemic symptoms and comprised 567 women. Follow‐up data were collected for at least 1 year, with most data gathered over 5 years (Merz et al. 1999).

Standard, resting 12‐lead ECG was performed at baseline enrollment in the parent study with an all‐digital recording system and analyzed as previously described (Merz et al. 1999; Pepine et al. 1994). ECG data included seven variables: RHR, PR interval duration, QRS interval, corrected QT interval (at 60 bpm) as continuous variables; underlying rhythm (normal sinus, sinus bradycardia, any tachycardia, atrial fibrillation/other), heart block/conduction delays (none, any left block, any right block, other), and abnormal ST‐segment/T‐wave (normal, any ST/T change, strain/other). Incident MACE was defined as the first occurrence of angina hospitalization, MI, stroke, revascularization (CABG or PTCA), CHF, or all‐cause death.

### 2.1 Statistical Analysis

Statistical analyses were conducted with SAS 9.4 (Cary, NC, USA). Descriptive statistics were summarized via means and frequencies for continuous and categorical variables, respectively. Participant characteristics between MACE and non‐MACE groups were compared using *t*‐test for means and chi‐square tests for frequencies, with a *p*‐value threshold of < 0.05 indicating statistically significant differences. Time in days, from study enrollment (baseline) to time at incident MACE (first occurrence of any MACE outcome) censored on days to last follow up, was analyzed by Cox proportional hazards analysis. A logarithmic transformation to the PR interval variable was applied in our Cox regression model to normalize its skewed distribution. Consequently, the model fit improved, and the assumptions of the Cox proportional hazards model were better satisfied. Seven ECG indices and 18 covariates (Table 1) were tested with stepwise backward selection and bootstrapping with 200 iterations. Predictors having *p*‐values < 0.15 and bootstrapped probability of 70% or greater were retained. The final model included two ECG indices (RHR and presence of arrhythmia) and five covariates (race, CHF, depression, chronic renal dysfunction, and use of nitrates within 24 h prior to ECG). Any predictors with *p*‐values < 0.05 were considered to have a statistically significant impact on the risk of time to MACE events. A post hoc power analysis was conducted for Cox survival using standard methods (Schoenfeld 1983).

**TABLE 1:** Predictors included in Cox regression model.

ECG predictors	Covariates
Corrected QT interval (QTc) QRS interval Intraventricular conduction delay (block) ST‐T wave abnormalities (Log‐transformed) PR interval Underlying cardiac rhythm **Resting heart rate (RHR)**	Age BMI Waist circumference Coronary flow reserve Menopause History of hypertension History of dyslipidemia History of smoking History of type 2 diabetes Peripheral vascular disease Calcium channel blockers 24 h prior ECG Beta blockers 24 h prior ECG ACE inhibitors 24 h prior ECG Lipid‐lowering statins 24 h prior ECG History of chronic renal dysfunction **Race** **Congestive heart failure** **History of depression** **Nitrates 24 h prior ECG**

*Note:* Variables with underscores were included in the final model and variables with bold font were statistically significant predictors in the final model.

## 3 Results

### 3.1 Participant Characteristics

The mean age of the participants was 55.6 years (range, 21–86) with nearly 17% self‐reported African Americans. Hispanic women (~2%) were under‐represented due to the WISE study's English language inclusion requirement (see Table 2). Most women in the sample self‐reported as non‐Hispanic White, overweight to obese and postmenopausal with positive histories of hypertension and smoking. Compared to women who did not experience a MACE event, those with positive MACE, on average, had significantly higher waist circumference, were more likely to self‐report race as African American/other, had histories of hypertension, type 2 diabetes, CHF, and depression, and were more likely to be taking statins, calcium channel blockers, and nitrates within 24 h prior to ECG.

**TABLE 2:** Sample characteristics.

Characteristic	Suspected INOCA survival analysis model (*n =* 481) mean ± SD, Freq (%)	No MACE group (*n =* 316) mean ± SD, Freq (%)	MACE group (*n =* 165) mean ± SD, Freq (%)	*p* (MACE vs. non‐MACE)
Age (years)	55.7 ± 10.7	55.3 ± 10.4	56.5 ± 11.3	0.2261
BMI (kg/m^2^)	30.0 ± 6.9	29.7 ± 6.8	30.4 ± 7.0	0.3035
Waist circumference (in)	36.4 ± 6.6	35.9 ± 6.4	37.5 ± 6.8	**0.0142**
CFR (ratio)	2.6 ± 0.8	2.6 ± 0.8	2.6 ± 0.8	0.997
Race				**0.0394**
White, Non‐Hispanic	398 (82.7)	271 (85.8)	127 (77.0)	
AA Non‐Hispanic	80 (16.6)	44 (13.9)	36 (21.8)	
Hispanic/other	3 (0.6)	1 (0.3)	2 (1.2)	
Hypertension	256 (53.2)	155 (49.1)	101 (61.2)	**0.0112**
Dyslipidemia	209 (43.5)	128 (40.5)	81 (49.1)	0.1384
Type 2 diabetes	78 (16.2)	43 (13.6)	35 (25.2)	**0.0359**
CHF	22 (4.6)	0	22 (13.3)	**< 0.0001**
CRD	12 (2.5)	9 (2.8)	3 (1.8)	0.7887
PVD	34 (7.1)	16 (5.1)	18 (10.9)	0.0574
Depression	135 (28.1)	74 (23.4)	61 (37.0)	**0.0017**
Smoking				0.6285
Former	156 (32.5)	91 (31.3)	57 (34.8)	
Never	231 (48.1)	157 (49.7)	74 (45.1)	
Current	93 (19.4)	60 (19.0)	33 (20.1.4)	
Menopause				0.0996
Pre	92 (19.1)	66 (20.9)	26 (15.8)	
Post	340 (70.7)	212 (67.1)	128 (77.6)	
Peri	41 (8.5)	32 (10.1)	9 (5.5)	
Other/unknown	8 (1.7)	6 (1.9)	2 (1.2)	
*Medication use within 24 h prior to ECG*				
Beta blockers				0.7428
Yes	122 (25.4)	77 (24.4)	122 (25.4)	
No	308 (64.0)	204 (64.6)	308 (64.0)	
Unknown	51 (10.6)	35 (11.1)	51 (10.6)	
Statins				**0.0181**
Yes	94 (19.5)	52 (16.5)	42 (25.5)	
No	387 (80.5)	264 (83.5)	123 (74.5)	
CCB				**0.0027**
Yes	88 (18.3)	44 (13.9)	44 (26.7)	
No	343 (71.3)	238 (75.3)	105 (63.6)	
Unknown	50 (10.4)	34 (10.8)	16 (9.7)	
ACEI				0.1056
Yes	68 (14.1)	37 (11.7)	31 (18.8)	
No	362 (75.3)	245 (77.5)	117 (70.9)	
Unknown	51 (10.6)	34 (10.8)	17 (10.3)	
Nitrates				**0.0181**
Yes	85 (17.7)	40 (12.7)	45 (27.3)	
No	345 (71.7)	241 (76.3)	104 (63.0)	
Unknown	51 (10.6)	35 (11.1)	16 (9.7)	

*Note:* Bold values: *p* < 0.05.

Abbreviations: ACEI, angiotensin‐converting enzyme inhibitors; CCB, calcium channel blockers; CFR, coronary flow reserve; CHF, congestive heart failure; CRD, chronic renal dysfunction (> 1.5 creatinine); PVD, peripheral vascular disease.

### 3.2 ECG Summary

Among women with MACE, the average values for RHR, PR, QRS, and QTc intervals were 71.9 bpm, 0.161 s, 0.086 s, and 432.18 ms, respectively (see Table 3). The most common abnormal categorical ECG findings were the presence of any ST‐ or T‐wave abnormality (26.0%), sinus bradycardia (20.8%), and 11.9% had ‘other’ heart blocks that were categorized as nonspecific, bifasic, or other. Participants in the non‐MACE group had lower RHR, shorter PR and QTc intervals, and had a higher prevalence of normal rhythm and ST waves; yet had a slightly higher frequency of heart blocks as compared to the MACE group.

**TABLE 3:** Baseline ECG characteristics.

Predictor	Suspected INOCA, analyzed (*n* = 481)	No MACE group (*n* = 316)	MACE group (*n* = 165)	Female normal ranges^b^
PR interval (s)	0.16 ± 0.03	0.156 ± 0.025	0.161 ± 0.028	0.12–0.20 s.
QRS interval (s)	0.09 ± 0.02	0.088 ± 0.016	0.86 ± 0.016	0.08–0.12 s.
QTc^a^ interval (ms)	427.51 ± 28.67	425.08 ± 27.72	432.18 ± 29.93	360–460 ms
Resting HR (bpm)	70.48 ± 13.13	69.74 ± 12.19	71.90 ± 14.71	60–100 bpm
Underlying rhythm				N/A
Normal	369 (76.7)	246 (77.8)	123 (74.5)	
Sinus bradycardia	100 (20.8)	66 (20.9)	34 (20.6)	
Any tachycardia	11 (2.3)	4 (1.3)	7 (4.2)	
Afib/other	1 (0.2)	0 (0)	1 (0.6)	
Block/delay				N/A
None/normal	392 (81.5)	256 (81.0)	136 (82.4)	
Any left block	20 (4.2)	11 (3.5)	9 (5.5)	
Any right block	12 (2.5)	8 (2.5)	4 (2.4)	
Other	57 (11.9)	41 (13.0)	16 (9.7)	
ST‐T waves				N/A
Normal	345 (71.7)	236 (74.7)	109 (66.1)	
Any ST or T change	125 (26.0)	73 (23.1)	52 (31.5)	
Strain/other	11 (2.3)	7 (2.2)	4 (2.4)	

^a^ QTc: Corrected QT Interval at 60 beats per minute.

^b^ Rautaharju et al. (2023).

### 3.3 MACE Outcome Summary

**FIGURE 1:**
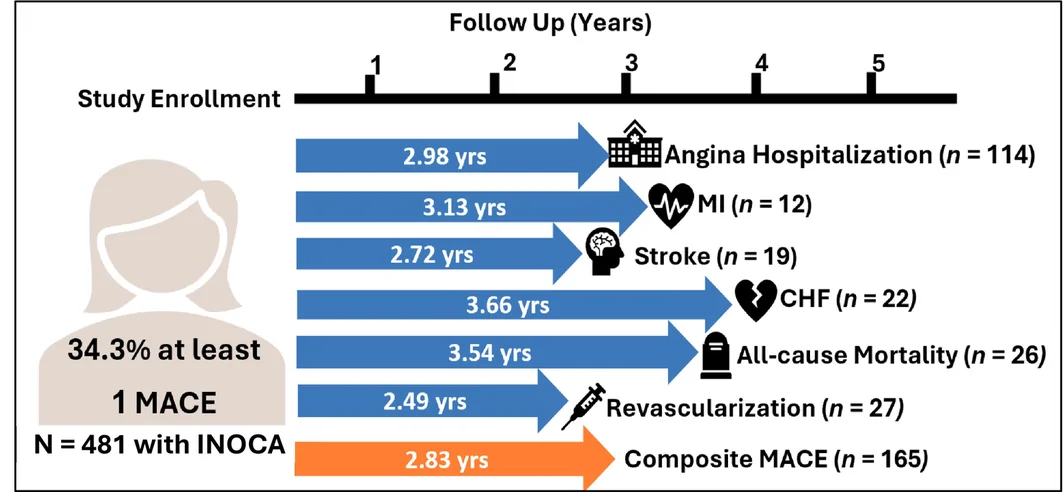
Mean time to event in years. CHF, congestive heart failure; MACE, major adverse cardiac event; MI, myocardial infarction.

Among the 65.7% of participants alive at last follow up, the mean follow‐up time was 5.25 years (see Table 4). Of the 481 women analyzed, 165 (34.3%) experienced at least one MACE event, averaging 1034.1 days (2.83 years) from enrollment. The most common MACE event was angina‐related hospitalization (*n* = 114) within an average of 2.98 years from study enrollment. Figure 1 shows a complete summary of MACE events.

**TABLE 4:** MACE occurrence and event times, in days.

Outcome	Complete data for survival analysis (*N* = 481) occurrence (mean ± SD)
MACE composite	165 (1034.1 ± 780.6)
Angina hospitalization	114 (1087.5 ± 765.2)
MI	12 (1145.0 ± 740.1)
Stroke	19 (995.1 ± 684.8)
Revascularization interventions	
CABG	11 (936.7 ± 871.3)
PTCA	37 (952.5 ± 916.0)
CHF	22 (1336.3 ± 866.3)
All‐cause death	26 (1291.4 ± 819.9)
Alive, time to last follow up	455 (1585.9 ± 1021.8)

Abbreviations: CABG, coronary artery bypass graft; CHF, congestive heart failure; MACE, major adverse cardiac events; MI, myocardial infarction; PTCA, percutaneous coronary transluminal angioplasty.

### 3.4 Cox Proportional Hazard Model

In the final model containing two ECG indices and five clinical covariates (Table 5), RHR was independently associated with a 1.018‐fold increased hazard of incident composite MACE among women with INOCA (HR: 1.018, 95% CI: 1.0–1.035). Secondarily, other statistically significant predictors in the final model included a history of CHF and nitrate use 24 h before ECG (both *p* < 0.0001), as well as race and a history of depression (*p* = 0.0114, *p* = 0.0013, respectively).

**TABLE 5:** Survival analysis model of incident composite MACE outcome.

Variables	Categories	Hazard ratio	95% CI		*p*
Race	Black	1.689	0.073	12.73	**0.** **0114**
	Other	3.041	1.15	2.485	
Resting heart rate		1.018	1	1.035	**0.** **0348**
CHF	Yes	4.688	2.92	7.525	**< 0.** **0001**
Depression	Yes	1.731	1.24	2.419	**0.** **0013**
Nitrate use	Yes	2.258	1.58	3.232	**< 0.** **0001**
CRD	Yes	0.274	0.09	0.888	0.0963
	Missing	0.868	0.12	6.29	
Underlying Rhythm	Sinus bradycardia	1.388	0.83	2.32	0.01729
	Any tachycardia	1.954	0.80	4.748	
	Afib/other	1.489	0.19	11.593	

*Note:* Bold values: *p* < 0.05.

Abbreviations: CHF, congestive heart failure; CRD, chronic renal disease.

### 3.5 Post Hoc Power Calculation

To assess the adequacy of our sample size and the strength of our findings, we calculated the detectable hazard ratio based on the observed event rate (165/481). Assuming a two‐tailed alpha level of 0.05, a power of 80%, and using the standard normal deviates for α (Zα = 1.960) and β (Zβ = 0.8416), we determined that the study was powered to detect a hazard ratio of at least 1.58.

## 4 Discussion

Our primary finding identified baseline RHR as an independent predictor of MACE among women with INOCA in a multivariate model, conferring a nominal yet statistically significant 1.02‐fold increased hazard effect (*p* = 0.0348, 95% CI 1.0–1.035). This relation was observed with a slightly higher mean RHR (71.9 bpm, range 57.19–86.61) among those experiencing at least one MACE event as compared to those who did not (69.7 bpm, range 57.55–81.93). Our secondary findings included the identification of other clinical predictors of MACE in this unique population. Our bootstrapping procedure identified the predictors with at least 70% likelihood of being included in a time‐to‐MACE model; the subsequent Cox model containing these variables revealed the following independent predictors: race (*p* = 0.0114), CHF (*p* < 0.0001), depression (*p* = 0.0013), and use of nitrates 24 h prior to ECG (*p* < 0.0001).

Comparisons of our findings to the INOCA literature are sparse. We observed an estimated 34% composite event rate with most incident events occurring an average of 2.83 years from baseline. By comparison, a Denmark‐based study, with follow‐up period of 7.5 years, observed an 11.9% event rate for symptomatic women with either diffuse or normal coronary arteries (most comparable to INOCA), followed by an 18% event rate for women with between 1‐and‐3‐vessel obstructive disease (Jespersen et al. 2012).

Members of our group have previously reported that RHR was a significant predictor of CHF in this same WISE INOCA cohort (Leong et al. 2021). Otherwise, reports are limited to the influence of stress wall abnormalities with positive ECG findings or peak HR obtained during stress testing (Ranasinghe et al. 2023; Cortigiani et al. 2021) in the INOCA population. Our results are consistent with other literature demonstrating RHR variation as an indisputably significant independent predictor of CAD, stroke, sudden death and mortality in people with subclinical and overt cardiovascular disease, even after controlling for cardiovascular risk factors (O'Neal et al. 2017; Lonn et al. 2014; Munzel et al. 2019; Olshansky et al. 2016; Zhang et al. 2016). Notably, our observed MACE‐associated RHR values are within the normal range (60–100 bpm) in the context of a small effect size (HR ~1.2) and narrow confidence interval. It has been established that increased hazards can occur with RHR in the normal range, as supported by Benetos et al. (1999). They evaluated baseline resting heart rate in 19,386 French adults from the general population who were followed for 18.2 years, showing an independent relationship with mortality for RHR varying from 60 to ≥ 100 bpm (Benetos et al. 1999). Our observed mean RHR (71.9 bpm) in the MACE group vs. 69.7 bpm in the non‐MACE group is consistent with multiple reports of significant differences in MACE risk at the 70 bpm cutoff (O'Neal et al. 2017; Lonn et al. 2014; Munzel et al. 2019; Zhang et al. 2016). Our small effect size (hazard ratio 1.018) is also consistent with meta‐analyzed effect estimates ranging 1.05–1.25 and having narrow 95% CIs for CAD, stroke, and mortality among > 1.2 million adults from 45 prospective cohort studies (Zhang et al. 2016). Among women, the average elevated MACE risk was 1.13 for every 10 bpm increase in RHR (Zhang et al. 2016). Reflecting on relevant sex differences, female sex and younger age are independently associated with RHR (Munzel et al. 2019). There is an established trend of females having an average constant of 7–9 bpm higher RHR as compared to age‐matched men, and without age‐related change (Munzel et al. 2019). However, the apparent impact of their average higher RHR on MACE events may be attenuated compared to men. Analysis of UK BIOBANK data revealed slightly lower risk of CV‐ and all‐cause‐mortality with each 10 bpm increase in HR in women compared to men (14%–19% vs. 17%–22%, respectively) (Raisi‐Estabragh et al. 2020). Similarly, an evaluation of Copenhagen City Heart Study (Jensen et al. 2012) revealed that when comparing those with heart rate > 80 to < 65 bpm, the survival difference for men was a loss of 4.7 years and for women, a loss of 3.6 years.

While kidney dysfunction and the presence of an abnormal baseline heart rhythm were identified based on bootstrapping metrics and thus included in the final model, they were not independently predictive of the outcome (*p* = 0.0963, *p* = 0.1729, respectively). However, their inclusion in the final model reinforces known relationships and potential collinearity. The presence of kidney disease is a well‐established independent risk factor for mortality and MACE events. The kidneys indirectly regulate heart rate via the renin‐angiotensin‐aldosterone feedback loop. Analyses of large population cohorts concluded that RHR is also highly predictive of glomerular filtration rate and the presence of kidney disease (Tsai et al. 2023; Widatalla et al. 2024), making it a potentially useful marker for kidney disease prevention (Abdin and Bohm 2023). The presence of arrhythmias also has well‐established findings as an independent risk factor for mortality with clear physiological ties to RHR (Olshansky et al. 2016). RHR is largely influenced by vagus nerve conduction to the pacemaker cells within the sinus node (Olshansky et al. 2016). Various compensatory and pathologic mechanisms (predominantly involving sympathetic tone) can lead to arrhythmias which adversely impact RHR (Lonn et al. 2014; Olshansky et al. 2016). Ventricular arrhythmias have the highest risk of sudden cardiac death (Lonn et al. 2014). In our sample, sinus bradycardia constituted the largest proportion of baseline arrhythmias with approximately 21% among both MACE and non‐MACE groups; atrial fibrillation and any tachycardia represented approximately 6% of the sample and were slightly overrepresented in the MACE group.

**FIGURE 2:**
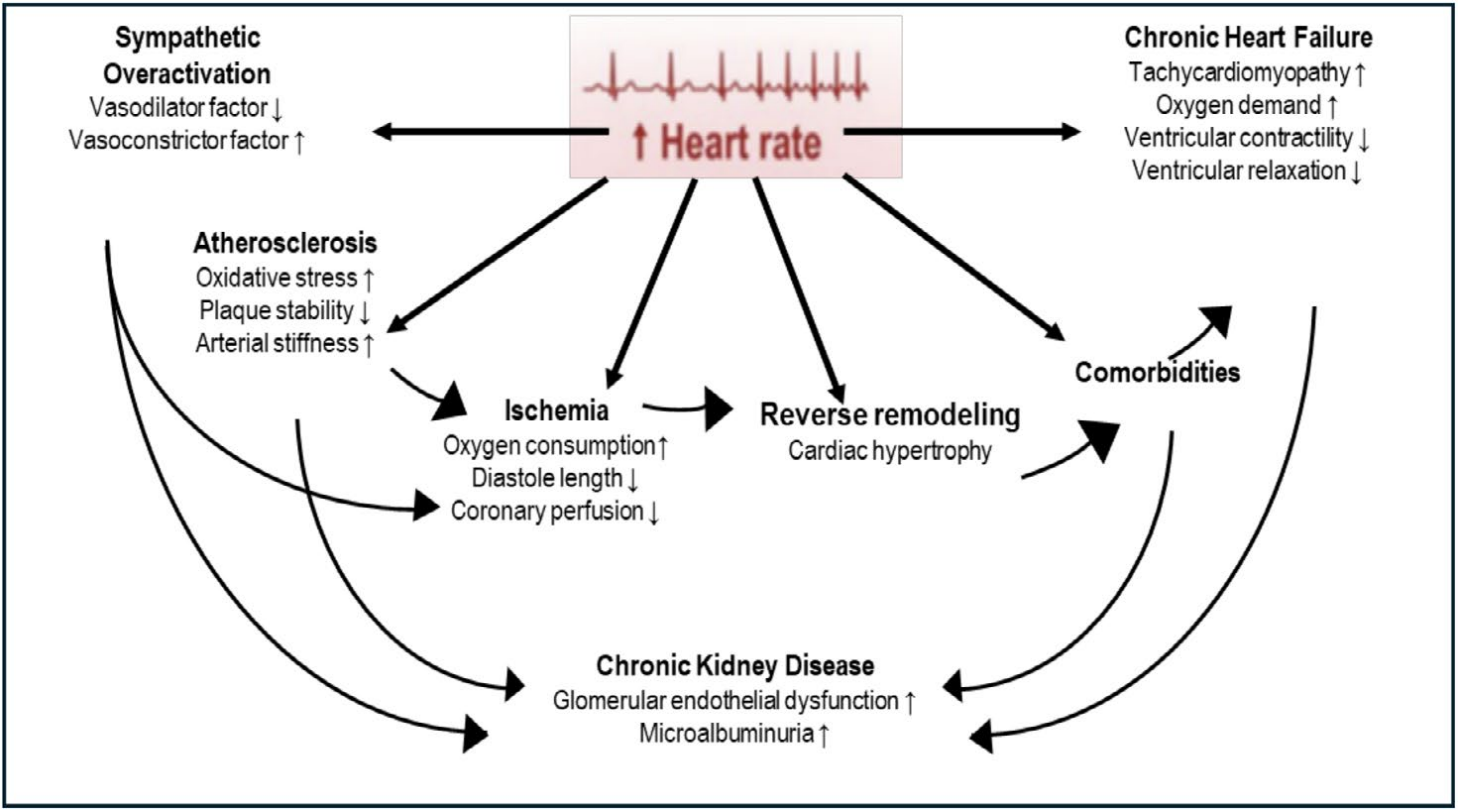
Pathophysiologic mechanisms by which increased RHR impacts cardiovascular ischemia and comorbidities (Adapted with requested permission from Abdin and Bohm (2023), Pathophysiological effects of heart rate on the cardiovascular disease continuum and chronic kidney disease, Journal of the American Heart Association Cardiovascular and Cerebrovascular Disease, Wiley Periodicals LLC on behalf of the American Heart Association Inc.).

The proposed mechanism by which RHR could impact MACE and mortality primarily centers around aberrant sympathetic activity and tone, as discussed at length elsewhere (Cortigiani et al. 2021; Lonn et al. 2014; Olshansky et al. 2016). Briefly, increased sympathetic activity and tone lead to increased high entry heart rate, promoting myocardial oxygen demand, sheer endothelial stress, accelerated atherosclerosis and plaque rupture, and life‐threatening arrhythmias (Cortigiani et al. 2021; Lonn et al. 2014; Olshansky et al. 2016). As depicted in Figure 2, the pathophysiological effects of elevated heart rate are systemic within cardiovascular and cardiometabolic processes, with high RHR being independently associated with hypertension, diabetes and other metabolic syndromes (Lonn et al. 2014). Therefore, the risk effects of RHR are likely mediated through comorbidity development and may represent an early risk marker.

Our lack of findings for QT and T‐wave related ECG indices was somewhat unexpected, as these were the most common independent ECG predictors of MACE in prior WISE analyses with different female INOCA subsets or outcomes (Triola et al. 2005; Mehta et al. 2017; Ranasinghe et al. 2023) as well as in other non‐INOCA studies (Brenyo and Zareba 2011; Kashani and Barold 2005; Kwok et al. 2016; O'Neal et al. 2017; Welten, Elders, et al. 2023; Welten, van der Heijden, et al. 2023; Thygesen et al. 2018), and in a single MINOCA study to date (Nordenskjold et al. 2018), many of which did not evaluate sex differences. Specifically reflecting upon inconsistent findings between our work and prior ECG‐related analyses of WISE participants, potential reasons for differential results most likely involve design or analytic heterogeneity. Triola et al. (2005) likely lacked sufficient power given their analyses of initial enrollees for WISE (*N* = 143). Authors also conducted deep phenotyping of sophisticated, lead‐specific ECG parameters and only evaluated three MACE outcomes (death, CHF, non‐fatal MI) as compared to our six‐outcome composite. Results reported by Mehta et al. (2017) were comparatively robust (*N* = 904 and 5‐year follow‐up) but included only women with INOCA and preserved ejection fraction, comparing them to women with obstructive disease (not comparable to our design), and only evaluated sudden cardiac death and mortality endpoints. ECG differences among people with heart failure based on preserved vs. reduced ejection fraction (Alhadramy et al. 2023), including RHR (Bristow and Altman 2017) have been reported but also vary, suggesting that our inclusion of women with HF regardless of EF status could contribute to the differences in findings. There is a noted lack of sex differences in this literature for comparison as well. Finally, we least expected our results to be consistent with that of Ranasinghe et al. (2023), as they evaluated composite MACE outcomes among WISE participants specifically having an absence of ST change (termed negative ECG) and either positive or negative evidence of wall motion abnormalities during dobutamine stress testing. This study was most heterogenous in comparison. Our differential findings do not appear to be a product of low variability in aberrant ECG indices, as our observed frequency of QRS‐ and T‐ related alterations in this female INOCA sample aligns with that of others (Reynolds et al. 2021), despite some reports of ST changes being less common in women compared to men (Ezaki et al. 2010; Kittnar 2023).

### 4.1 Limitations

Due to missing ECG and MACE data, we excluded 86 participants (approximately 15% loss) to complete our analysis, yet we retained adequate power. Stress and dobutamine ECG testing were excluded from our data as the primary objective was to identify ECG baseline changes in the absence of stress provocation. The application of our findings is limited to women, who represent the largest proportion of INOCA cases and health disparities related to coronary ischemia (Mehta et al. 2022). We evaluated 25 variables including medication use, yet other unstudied factors may have contributed to MACE outcomes. Some events may be attributable to non‐cardiac deaths. As RHR was a key predictor, it would have been ideal to evaluate heart rate variability and 24‐h blood pressure as well; however, such data were lacking in the WISE study.

## 5 Conclusion

We demonstrate evidence for the independent role of RHR in predicting earlier time to MACE among women with INOCA, wherein those with CHF, depression, and history of nitrate use as well as those of African American ancestry appear to be at highest risk. Future biomarker research on INOCA and patient outcomes, addressing stated limitations, is crucial for advancing precision cardiovascular health strategies for women.

## Author Contributions

**Jennifer R. Dungan:** conceptualization, methodology, writing – original draft preparation, writing – review and editing, visualization, supervision, project administration, and funding acquisition. **Yasmeen K. Taha:** conceptualization, methodology, and writing – review and editing. **Qinglin Pei:** methodology, software, validation, formal analysis, writing – review and editing, and visualization. **Michael T. Weaver:** methodology, software, formal analysis, data curation, and writing – review and editing. **Michael J. Tavormina:** writing – review and editing. **Steven E. Reis:** writing – review and editing. **Eileen M. Handberg:** writing – review and editing. **C. Noel Bairey Merz:** resources, writing – review and editing, and funding acquisition. **Carl J. Pepine:** resources, writing – review and editing, and funding acquisition.

## Funding

This work was partially supported by the National Institute of Aging (No. R21 AG077715‐01A1) and via contracts from the National Heart, Lung and Blood Institutes, Nos. N01‐HV‐68161, N01‐HV‐68162, N01‐HV‐68163 and N01‐HV‐68164, and grants from the Gustavus and Louis Pfeiffer Research Foundation, Denville, New Jersey (no number), The Women's Guild of Cedars‐Sinai Medical Center, Los Angeles, California (no number), The Ladies Hospital Aid Society of Western Pennsylvania, Pittsburgh, Pennsylvania (no number) and QMED Inc., Laurence Harbor, New Jersey (no number). This work is solely the responsibility of the authors and does not necessarily represent the official views of the National Heart, Lung, and Blood Institute, the National Institutes of Health, or the US Department of Health and Human Services.

## Ethics Statement

The study was conducted according to the guidelines of the Declaration of Helsinki, and approved by the Institutional Review Board of the University of Florida (Protocol #IRB201501099, July 18, 2022) using data from participants who consented for secondary analysis of their anonymized, de‐identifiable data for cardiac research purposes who participated in the original Women's Ischemia Syndrome Evaluation (WISE) study biobank (University of Florida Protocol #IRB201501099).

## Conflicts of Interest

The following authors report no conflicts of interest: Y.K.T., M.T.W., E.M.H., and C.J.P. The following authors disclose the following relationships; however, these entities had no role in the design, execution, interpretation, or writing of the project: C.N.B.M. Consulting: IRHTYM.

## Data Availability

The data presented in this study are openly available in NIH/NHLBI's BioLINCC repository at URL: https://biolincc.nhlbi.nih.gov/studies/wise/, accession reference number HLB00490507a (accessed on June 29, 2023).
